# Very Prolonged Treatment with Albendazole of a Case of Disseminated Abdominal Cystic Echinococcosis

**DOI:** 10.3390/tropicalmed8090449

**Published:** 2023-09-15

**Authors:** Carola Buscemi, Cristiana Randazzo, Paolo Buscemi, Rosalia Caldarella, Martina Lombardo, Silvio Buscemi

**Affiliations:** 1 Unit of Internal Medicine, V. Cervello Hospital, I-90100 Palermo, Italy; carola.buscemi@unipa.it; 2 Dipartimento di Promozione della Salute, Materno-Infantile, Medicina Interna e Specialistica di Eccellenza (PROMISE), University of Palermo, I-90100 Palermo, Italy; rosalia.caldarella@unipa.it (R.C.); martina.lombardo05@unipa.it (M.L.); silvio.buscemi@unipa.it (S.B.); 3 Unit of Clinical Nutrition, AOU Policlinico “P. Giaccone”, I-90100 Palermo, Italy; 4 Postgraduate School in Radiology, University of Palermo, I-90100 Palermo, Italy; paolo.buscemi@community.unipa.it; 5 Unit of Laboratory Medicine, AOU Policlinico “P. Giaccone”, I-90100 Palermo, Italy

**Keywords:** albendazole, *Echinococcus* granulosus, disseminated cystic echinococcosis, cystic echinococcosis, liver cysts

## Abstract

Cystic echinococcosis is a zoonosis caused by the ingestion of food or water contaminated by *Echinococcus* eggs. E. granulosus is the most common causative agent of cystic echinococcosis that still has a relevant incidence in Italy, especially on the islands of Sicily and Sardinia. We report the case of a 64-year-old man with disseminated abdominal cystic echinococcosis (liver, spleen, peritoneum). The patient was asymptomatic and non-eligible for surgical treatment. Treatment with albendazole 400 mg/twice daily was started in 2012 for 15 cycles (each cycle consisted of three 28-day treatments at 14-day intervals) over 10 years for a total of 1260 days of treatment. Serum anti-*Echinococcus* antibody titers and imaging (echography, TC) were evaluated to monitor the evolution of the disease. Imaging techniques documented the regression of all cyst lesions, but it was less evident for the peritoneal localizations that still are in follow-up. In this case, the prolonged treatment with albendazole was effective, safe and free of side effects. Until today, the patient displays a good clinical condition.

## 1. Introduction

Cystic echinococcosis is a zoonosis caused more frequently by the ingestion of food or water contaminated by the larvae of *Echinococcus* granulosus or multilocularis of the Taeniidae family [1]. In Italy, the annual incidence of cystic echinococcosis is 1.6 for 100,000 inhabitants [2], but higher incidences were reported especially in Sardinia and Sicily islands [3]. The abdominal disseminated echinococcosis has an even less frequent occurrence that, in most cases, is due to the symptomatic or silent peritoneal rupture of a hepatic *Echinococcus* cyst (HEC) [4]. Mebendazole and albendazole (ABZ) are the two anthelmintic drugs used for medical treatment, with the latter being acknowledged to have better performance and safety profile. Therapy with ABZ is recommended for uncomplicated abdominal HEC with a diameter <5 cm, disseminated infection and peri-interventional prophylaxis of secondary echinococcosis [5,6]. ABZ inhibits both microtubule assembly and the activity of helminthic fumarate reductase leading to cell death. The most frequent side effects of ABZ include abdominal pain, anemia, bone marrow suppression with cytopenia, alopecia, increased reversible serum concentrations of aspartate (AST) and alanine transaminase (ALT) and gamma-glutamyl transferase (GGT) that may occur due to drug toxicity or to parasite death [7]. It was reported that treatment with only ABZ is successful in about 40% of cases of hepatic HEC [8]. However, no clinical trial has evaluated the long-term efficacy and safety of ABZ in patients with disseminated abdominal cystic echinococcosis. We describe the case of a patient with disseminated abdominal echinococcosis who was successfully treated with cycles of ABZ therapy for ten years.

## 2. Case Description

Mr P.R., a 64-year-old bricklayer, had his first visit in 2012 at the diabetes outpatients section of the Internal Medicine department of the University Hospital Policlinico “P. Giaccone” of Palermo (Sicily, Italy). He had well-controlled type 2 diabetes on metformin treatment (2 g/day). Abdomen ultrasound (US) and CT examinations performed in 2011 demonstrated disseminated cystic echinococcosis with hepatic (*n* = 3), splenic (*n* = 1), and peritoneum localizations (*n* = 2) of the cysts. In particular, the exams showed a partially exophytic cystic lesion with signs of rupture along the lower hepatic margin of the VI segment. So, it was concluded that the HEC rupture occurred asymptomatically and was probably responsible for the abdominal dissemination of the *Echinococcus*. Given the patient’s job, it was hypothesized that the HEC rupture was a consequence of abdominal trauma. After the exclusion of pulmonary involvement by CT scan of the thorax, the medical and surgical team defined the patient as non-eligible for surgical treatment, and the drug treatment with ABZ (400 mg/twice daily for three cycles of 28 days each, with a 14-day pause) was undertaken following the doses and protocol in use in 2012. Cautiously, metformin was discontinued and replaced with basal insulin (8–12 IU/day) and diet.

**Figure 1:**
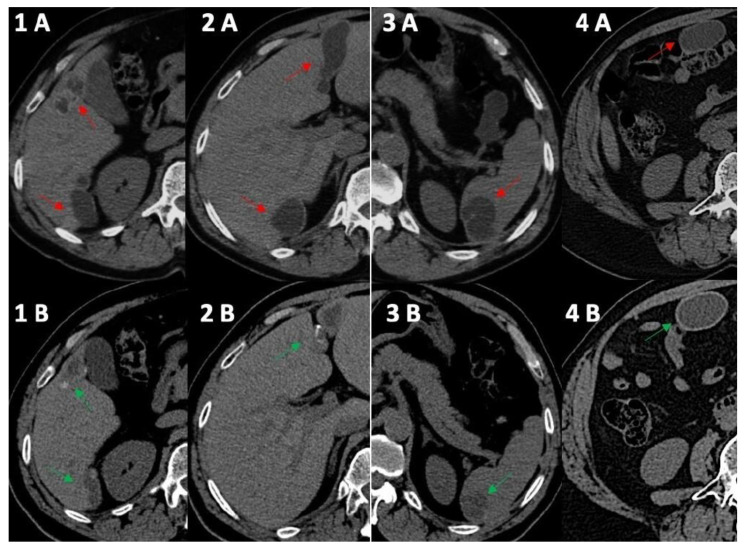
Computed tomography comparative imaging of some Echinococcus cysts in 2011 (A, red arrow) and in 2021 (B, green arrow). The cysts are located in liver segments 5 ((**1A**) 15 × 35 mm vs. (**1B**) 15 × 20 mm) and 6 (1–(**2A**) 41 × 45 mm vs. (**1B**) 10 × 36 mm), in segments 3–4 ((**2A**) 20 × 68 mm vs. **2B** 19 × 45 mm), in the upper pole of the spleen ((**3A**) 30 × 40 mm vs. (**3B**) 30 × 30 mm), and in the right inframesocolic space ((**4A**) 41 × 22 mm vs. 4B 40 × 26 mm). In 2021, CT scans (**1B**,**2B**,**3B**) showed some initial signs of cystic wall calcifications that were not present in 2011 (**1A**, **2A**, **3A**). Uncertainty persisted for the right inframesocolic cyst (**4B**), but in 2023 (Figure 2, #3), US demonstrated signs of intracystic degenerative content (Figure 2—n3).

**Figure 2:**
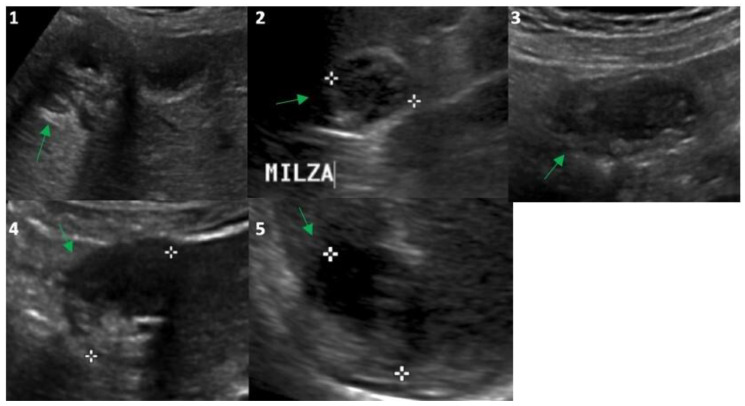
Ultrasound evaluation in 2023 of the *Echinococcus* cysts (green arrows) presented in Figure 1: segment 5 of the liver (frame (**1**); 25 mm), upper pole of the spleen (frame (**2**); 26 mm), right inframesocolic space (frame (**3**); 36 mm), segment 3–4 (frame (**4**); 30 mm) and 6 (frame (**5**); 30 mm) of the liver. White asterisks correspond to the points of the echo-calipers used to measure the size of the cysts.

A follow-up was started monitoring, in particular, anti-*Echinococcus* granulosus antibody titers (ELISA; regional reference center for diseases transmitted by arthropods—Sicily Region, Italy), US and CT imaging of the abdomen (Table 1; Figure 1 and Figure 2). Serological tests are problematic primarily for the diagnosis of cystic echinococcosis [9]; however, despite its low sensitivity, the ELISA assay is the best laboratory procedure, and we used serially detected antibodies against *Echinococcus* granulosus to improve the quality of follow-up to support our clinical strategy. Due to the slow but favorable response of the HEC, it was decided to continue the treatment with ABZ until a possible definitive regression of the disease was confirmed. No safety problems occurred until the second month of therapy with ABZ when increased serum concentrations of AST, ALT and GGT were observed (Table 1). Although it was hypothesized that the abnormal values of AST, ALT and GGT were due to the toxic effects of ABZ on *Echinococcus*, given the persistence of high hepatic blood tests after the second cycle of ABZ, it was decided to suspend the treatment and to adopt a “watch and wait” strategy for that year. 

In 2014, the anti-*Echinococcus* titers were still elevated, serum AST, ALT and GGT were normalized and imaging of the cysts suggested possible activity especially for the peritoneal localizations; therefore, treatment with ABZ was re-started and continued until 2021. Normal hepatic blood test results were observed until the end of treatment with ABZ in 2021 (Table 1) and a final (2021) hepatic FibroScan demonstrated normal stiffness values (May 2021, 2.8 kPa). We observed no significant change in the eosinophil count for the whole duration of the follow-up (Table 1), thus excluding a significant relationship between eosinophilia in this case of cystic echinococcosis [10]. Finally, at the end of 2021, the patient had received a total of 15 complete cycles with ABZ that equaled 1260 days of treatment. All cystic lesions progressively showed US [11] and CT signs of regression (Figure 1 and Figure 2) with some uncertainty for peritoneal lesions, which are still in follow-up in 2023. In particular, based on WHO-IWGE US classification [11], in 2023, all cysts were defined as inactive (CE4–5; Figure 2, frame 1, 2, 4, 5) with the exception of the peritoneal lesion that was considered transitional (CE3; Figure 2, frame 3).

Subsequent measurements of anti-*Echinococcus* antibody titers reported values of 1/200 or were undetectable. In fact, after treatment with ABZ, the patient displayed good health with adequate glycemic control and nutritional state (body weight at the last visit: 71.5 kg, +2.4 kg from 2021), including normal body composition (BIA-101 Akern, Italy) and bioelectrical phase angle (7.7°).

## 3. Discussion

According to the WHO, cystic echinococcosis is a Neglected Tropical Disease (NTD), relatively neglected by scientific research and public/private funding, compared to the magnitude of the health problem [12]. Cystic echinococcosis sometimes causes severe illness or death, especially when the clinical picture of the disseminated form occurs [13]. In that case, we have no clear indications about the most efficacious strategy to follow. As described in our case, the rupture of a hepatic cyst is the most frequent cause of disseminated abdominal echinococcosis. The rupture of a cyst is often a clinically dramatic event. In fact, data reported in the literature show that all cases of spontaneous intraperitoneal ruptured hepatic echinococcosis were treated by emergency surgery, except one case that was treated successfully by initial conservative measures, followed by elective radical surgery two months later [14]. Therefore, our case report was a particularly complex picture of disseminated echinococcosis following a clinically silent rupture of a hepatic cyst that had a favorable conclusion, demonstrating that long-term treatment with ABZ is possible, at least in the absence of symptoms due to mass effects. Our observation is in agreement with Zavoikin and colleagues who reported a follow-up of 117 patients with pulmonary echinococcosis in which ABZ administered from 3 months to 11 years was well tolerated and effective [15]. Also, cystic echinococcosis of the bone, which is a rare but very destructive form of the disease, requires chronic treatment. A recent European report by Cattaneo et al. [16] retrospectively identified 32 cases until 2018. All these patients underwent surgical treatment that was followed by lifelong treatment with albendazole (continuous treatment or on/off protocols). Overall, they report that the treatment was well tolerated and no patient had to stop treatment. However, to our knowledge, this is the first time that prolonged (10-year) treatment with ABZ has been reported in a case of disseminated abdominal cystic echinococcosis. It is also interesting to note that the treatment was well tolerated despite the patient being elderly and with a significant comorbidity such as diabetes. Given the evolution of serum concentrations of AST, ALT and GGT throughout the 10 years of treatment, we hypothesize that the initial high concentrations of these variables were a consequence of focal inflammatory damage due to the *Echinococcus* death and not to the direct hepatotoxic effect of ABZ, since it occurred only after the first two cycles of treatment. The peritoneal lesions produced some uncertainty about their evolution due to the fact that this site is possibly less reachable by the drug and imaging was less clear than for other classical localizations in the liver. However, the evolution of serum anti-*Echinococcus* antibody titers and the monitoring of cysts with echo imaging made it possible to conclude that ABZ was efficacious even in these problematic sites. Another point of interest is that after CT definition, the US method was an adequate technique of imaging for monitoring the evolution of cysts; therefore, after 2021, we decided to monitor the patient exclusively with echography, an inexpensive, easily repeatable, accurate and safe method [17]. Disseminated Echinoccoccosis is quite common and our case report demonstrates that treatment is complex despite effective, so we conclude that clinical trials with standardized diagnostic and therapeutic procedures are warranted to define the treatment of this parasitosis.

## 4. Conclusions

Cystic echinococcosis is a common endemic disease and disseminated abdominal cystic echinococcosis can cause severe illness or death. The case we describe suggests that long-term ABZ treatment might be a safe and effective treatment in patients with disseminated abdominal cystic echinococcosis. Clinical trials are warranted to define the most appropriate treatment of this parasitosis.

## Figures and Tables

**Table 1:** Evolution of laboratory tests and cycles of albendazole treatment from 2012 to 2021.

Year	2012	2013	2014	2015	2016	2017	2018	2019	2020	2021		
Month	Jul.	Mar.	Jul.	Oct.	Nov.	Oct.	Oct.	Oct.	Sep.	Gen.	May	Nov.
IgG anti-*echinococcus* (titers)	1/6400	1/6400	1/6400	1/3200	1/3200	1/1600	1/1600	1/800	1/800	1/400	-	1/200
ABZ cycles	2	-	2	1	1	2	1	1	2	1	1	1
AST (U/L)	140	198	58	19	27	23	28	22	26	24	16	20
ALT (U/L)	358	308	60	13	28	30	33	35	32	30	13	28
GGT (U/L)	179	268	101	32	19	29	27	32	30	28	25	34
White blood cells (n/mmc)	7920	7100		7430		7400		7500			10,100	
Eosinophils (%)	2.7	3.5		4.6		2.6		1.6			1.0	
Hemoglobin (g/dL)	12.7	12.0		12.2		12.4		12.3			12.5	

ABZ: Albendazole; AST: aspartate transaminase; ALT: alanine transaminase; GGT: gamma glutamyl transferase.

## Data Availability

No data to share besides those reported in this case report. Specific and motivated requests can be addressed to Silvio Buscemi, silvio.buscemi@unipa.it until 31 December 2024.
